# VCY mediates inhibition of PFV replication via interaction with the transcription activator Tas

**DOI:** 10.1128/jvi.00166-25

**Published:** 2025-06-03

**Authors:** Lin Jiang, Chunhua Han, Junshi Zhang, Guoqiang Li, Wentao Qiao, Juan Tan

**Affiliations:** 1 Key Laboratory of Molecular Microbiology and Technology, Ministry of Education, College of Life Sciences, Nankai University 616838 , Tianjin, China; University Hospital Tübingen, Tübingen, Germany

**Keywords:** prototype foamy virus, VCY, Tas, transcription

## Abstract

Prototype foamy viruses (PFVs) are complex retroviruses that establish long-term latent infections in hosts without causing disease, positioning them as potential safe gene transfer vectors. Understanding the host proteins involved in PFV replication and their interaction mechanisms may enhance gene transfer efficiency. However, only a few cellular proteins are known to influence PFV replication. In this study, transcriptomic analysis of PFV-infected HT1080 cells revealed significant downregulation of variable charge Y (VCY) mRNA, indicating its potential significance in regulating PFV replication. Overexpression of VCY significantly inhibits PFV replication, whereas VCY knockdown enhances viral replication, which can be reversed by reintroducing the VCY protein. VCY interacted with the Tas DNA-binding and transcriptional activation domains and interfered with its binding to the PFV long terminal repeat and internal promoter, inhibiting the transactivation function of Tas. The N-terminal region of VCY, which contains a nuclear localization signal, is essential for this function. Additionally, VCY suppresses bovine foamy virus (BFV) replication by impairing the transactivation activity of BFV Tas, suggesting a broad-spectrum inhibitory effect on FV replication. Collectively, our data elucidate the role of VCY in inhibiting PFV replication through transcriptional interference for the first time, providing valuable insights into viral latency and host interactions.

**IMPORTANCE:**

FVs can integrate into host chromosomes and are nonpathogenic in natural hosts or experimentally infected animals, making them safe and efficient gene transfer vectors. They establish lifelong latent infections without evident pathology in the host. To date, only a few host factors have been identified that affect PFV replication. In this study, we report that VCY inhibits PFV replication by modulating the function of the transcription activator Tas. Currently, there have been no studies examining the relationship between VCY and viruses, making this the inaugural report on its association with viral infection. Our data provide important insights into the role of VCY in PFV life cycle, which will aid in understanding the mechanisms underlying retroviral latent infection.

## INTRODUCTION

Foamy viruses (FVs), also known as spumaretroviruses, are the oldest retroviruses, belonging to the *Spumaretrovirinae* subfamily of the *Retroviridae* family (1). FVs are widely present in various hosts, including humans, felines, bovines, equines, and nonhuman primates (2–6). The prototype FV (PFV) was isolated from a patient with nasopharyngeal carcinoma in Kenya in the 1970s and is the best-characterized member of the FVs (7). In contrast to other retroviruses, PFV has two promoters in its genome: the long terminal repeat (LTR), which regulates the expression of the structural genes *gag*, *pol*, and *env*, and the internal promoter (IP), which regulates the expression of the accessory proteins Tas and Bet (1, 8).

FVs cause lifelong persistent infections, which are restricted by various host cell factors that inhibit viral replication. A variety of anti-FVs effectors target different steps of the virus replication cycle. The entry of PFV into host cells is impeded by interferon-induced transmembrane protein 3, which promotes the degradation of the envelope protein via the lysosomal pathway (9). Regarding the regulation of viral genome transcription, the antiviral activity of apolipoprotein B mRNA editing enzyme catalytic subunit 3G suppresses viral transcription through cytidine editing of the viral genome (10). Several antiviral factors that inhibit viral gene transcription are associated with its unique transactivator, Tas. For instance, N-myc interactor (Nmi) inhibits the transactivation effects of PFV Tas on the viral promoters LTR and IP by interacting with Tas, thereby sequestering it in the cytoplasm (11). The p53-induced RING-H2 protein (Pirh2) interacts with PFV Tas and promotes its degradation via the ubiquitin-proteasome pathway, thereby inhibiting Tas’s transactivation of the promoters and subsequently suppressing PFV replication (12). Serum/glucocorticoid regulated kinase 1 (SGK1) and PHD finger protein 11 impede Tas-induced transactivation and affect viral transcription (13, 14). During the viral release phase, human BST2 (hBST2, also referred to as tetherin) acts as a host restriction factor that obstructs the release of various enveloped viruses (15).

To identify new cell factors that inhibit PFV replication, we analyzed the differential mRNA expression profiles in PFV-infected cells and found that variable charge Y (VCY) expression was significantly downregulated, suggesting its important role during PFV infection. VCY is a gene family member specific to human testicular tissue and belongs to the variable charge X/Y (VCX/Y) family, which also includes members such as VCX-2r, VCX-8r, and VCX-10r (16). The VCY protein is localized in the cell nucleus and consists of 125 amino acids (17). Its N-terminal contains a nuclear localization signal (NLS) (19–36 aa), while its C-terminus features a region rich in glutamic acid composed of 10 amino acids (104–113 aa) (17, 18). Currently, based on residue composition, NLSs are primarily classified into classical NLS (cNLS), nonclassical NLS (ncNLS), and other types (19). The cNLS can be further divided into monopartite (MP) and bipartite (BP) signals (20). The NLS of VCY is classified as a BP, and the basic amino acids in the NLS are crucial (18, 21). Functionally, VCY is involved in the regulation of ribosomal protein assembly during spermatogenesis (22). Moreover, the VCX/Y family proteins exhibit high expression levels in lung cancer cells, identifying them as new cancer/testis antigens (CTAs) (23). However, no studies have specifically investigated the relationship between VCY and viruses. Therefore, whether VCY plays a role in PFV infection warrants further exploration.

In this study, we demonstrate that VCY interacts with the PFV transactivation factor Tas, thereby suppressing Tas’s activation of viral promoters LTR and IP, which inhibits PFV replication. Our data represent the first examination of the relationship between VCY and viruses, providing crucial insights into VCY’s function in the PFV life cycle and enriching our comprehension of retrovirus infection mechanisms.

## RESULTS

### Downregulation of *vcy* by PFV infection

**Fig 1:**
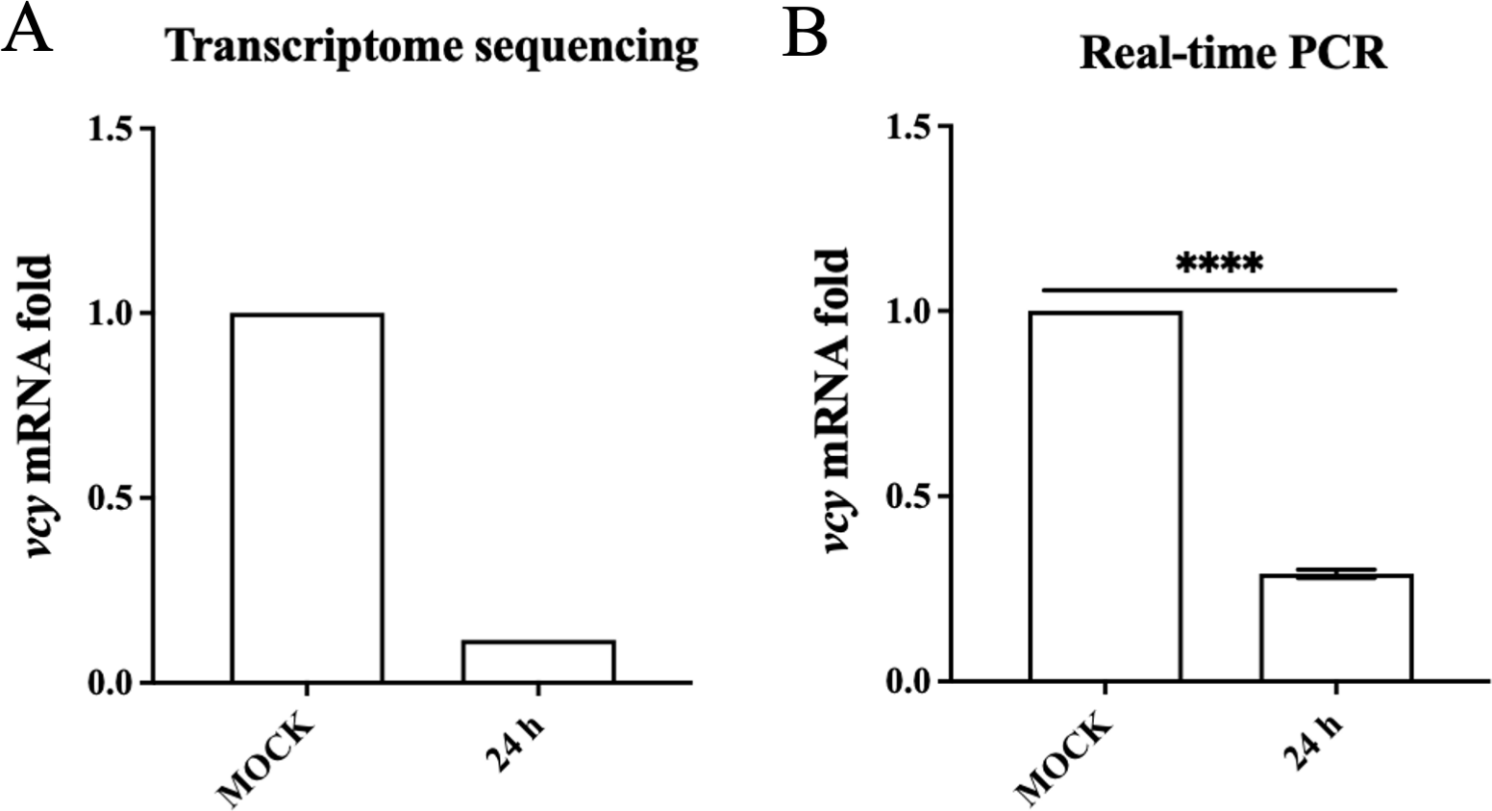
Downregulation of *vcy* by PFV infection. (**A**) The result of transcriptomic sequencing. (**B**) HT1080 cells were infected with PFV (MOI = 0.5) for 24 h, and *vcy* mRNA levels were measured by qRT-PCR. All the data are representative of three independent experiments with triplicate samples. (^****^P < 0.0001).

To identify cellular factors involved in PFV replication, we determined the transcriptomes of HT1080 cells infected with PFV for 24 h to assess changes in the mRNA levels of cellular genes. The results demonstrated significant alterations in the mRNA levels of 394 genes in PFV-infected cells after 24 h (13). Approximately 46 genes exhibited downregulated mRNA levels, among which the downregulation of *vcy* mRNA was the most significant, with a reduction of up to eightfold. However, no studies have focused on the interactions between VCY and viruses. Therefore, to explore VCY’s involvement in PFV replication, we first verified the results of transcriptome sequencing. The quantitative real-time PCR (qRT-PCR) results showed that compared with the uninfected group, the endogenous *vcy* mRNA level was significantly downregulated at 24 h after PFV infection (Fig. 1B), consistent with the results of transcriptome sequencing (Fig. 1A).

### Overexpression of VCY inhibits PFV replication

**Fig 2:**
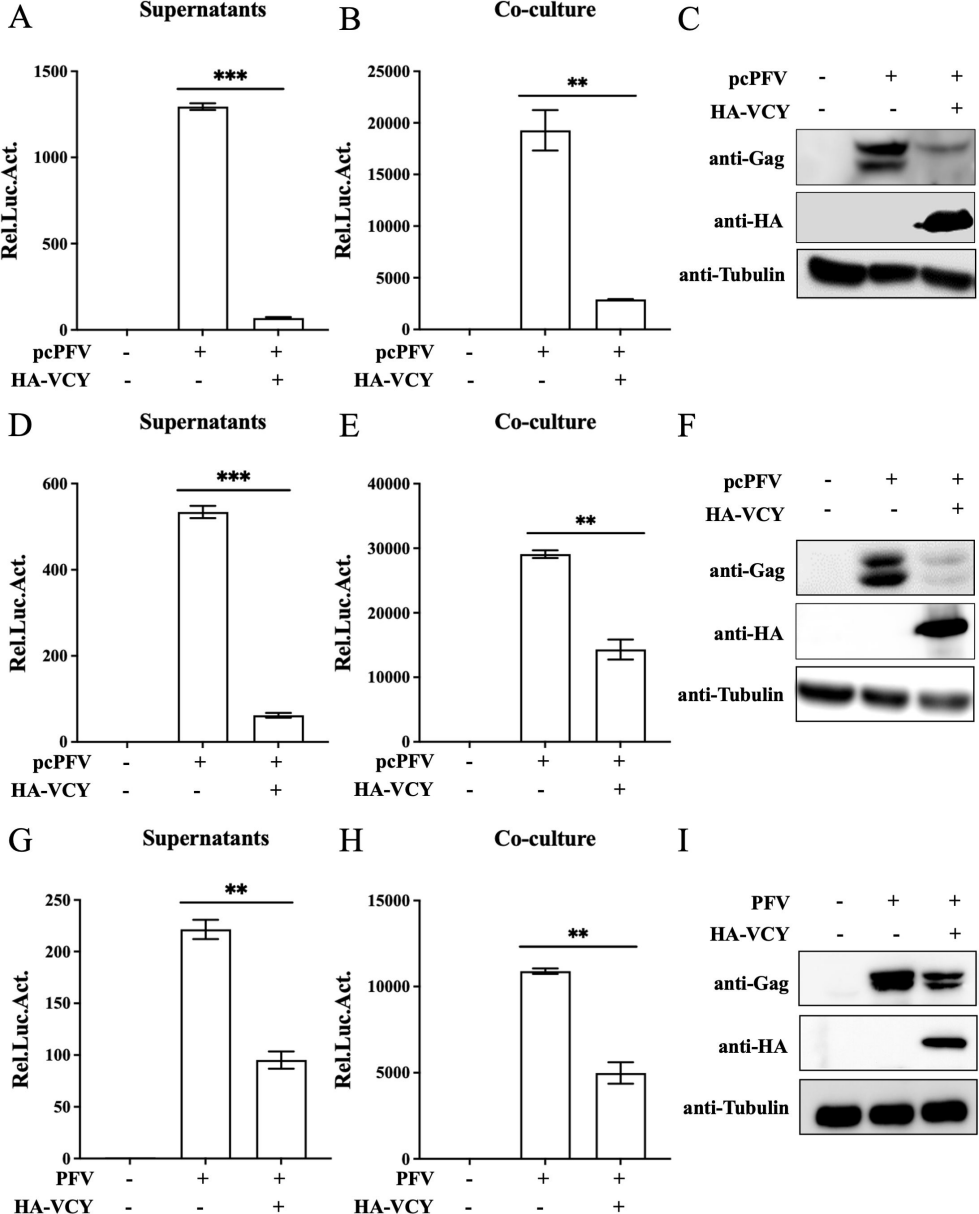
Overexpression of VCY inhibits PFV replication. (**A through C**) HEK293T cells or HT1080 cells (**D through F**) were transfected with pcPFV and HA-VCY or empty vector. After 48 h, 600 µL of supernatants (**A and D**) or 1/10 of infected cells (**B and E**) were co-cultured with PFVL cells to determine viral titers by luciferase assay. The rest of the infected cells were lysed for western blotting (**C and F**). (**G through I**) HT1080 cells were infected with PFV (MOI = 0.8) after transfected with HA-VCY or an empty vector for 8 h. After 40 h, 600 µL of supernatants (**G**) or 1/10 of infected HT1080 cells (**H**) were co-cultured with PFVL cells to determine viral titers by luciferase assay. (**I**) Western blotting was used to detect viral protein expression levels. All the data are representative of three independent experiments with triplicate samples. (^**^P < 0.01, ^***^P < 0.001).

To investigate the effect of VCY on PFV replication, we transfected full-length infectious clone pcPFV and plasmid HA-VCY in HEK293T cells. Forty-eight hours post-transfection, the culture supernatants and transfected HEK293T cells were incubated with the PFV indicator cell line (PFVL) for another 48 h, and luciferase activity was measured then. PFVL cells were created by transfecting baby hamster kidney-21 cells with a reporter plasmid containing a firefly luciferase gene driven by the PFV LTR promoter, ensuring that luciferase expression depends on the PFV transactivator Tas, with expression level directly proportional to the amount of Tas. Therefore, PFVL can be used to indicate PFV titer. The remaining transfected HEK293T cells were collected for western blotting analysis. The levels of both cell-free and cell-associated PFV were reduced by VCY overexpression (Fig. 2A through C). Similar results were observed in HT1080 cells (Fig. 2D through F). To further confirm the inhibitory effect of VCY on PFV, we transfected plasmid HA-VCY in HT1080 cell and infected with PFV virus stock solution. Compared to control cells, VCY-overexpressing cells significantly reduced both cell-free and cell-associated PFV levels (Fig. 2G and H). Additionally, it significantly reduced PFV Gag expression in transfected cells (Fig. 2I). These results further indicate that VCY inhibits PFV replication.

### Knockdown of endogenous VCY promotes PFV replication

**Fig 3:**
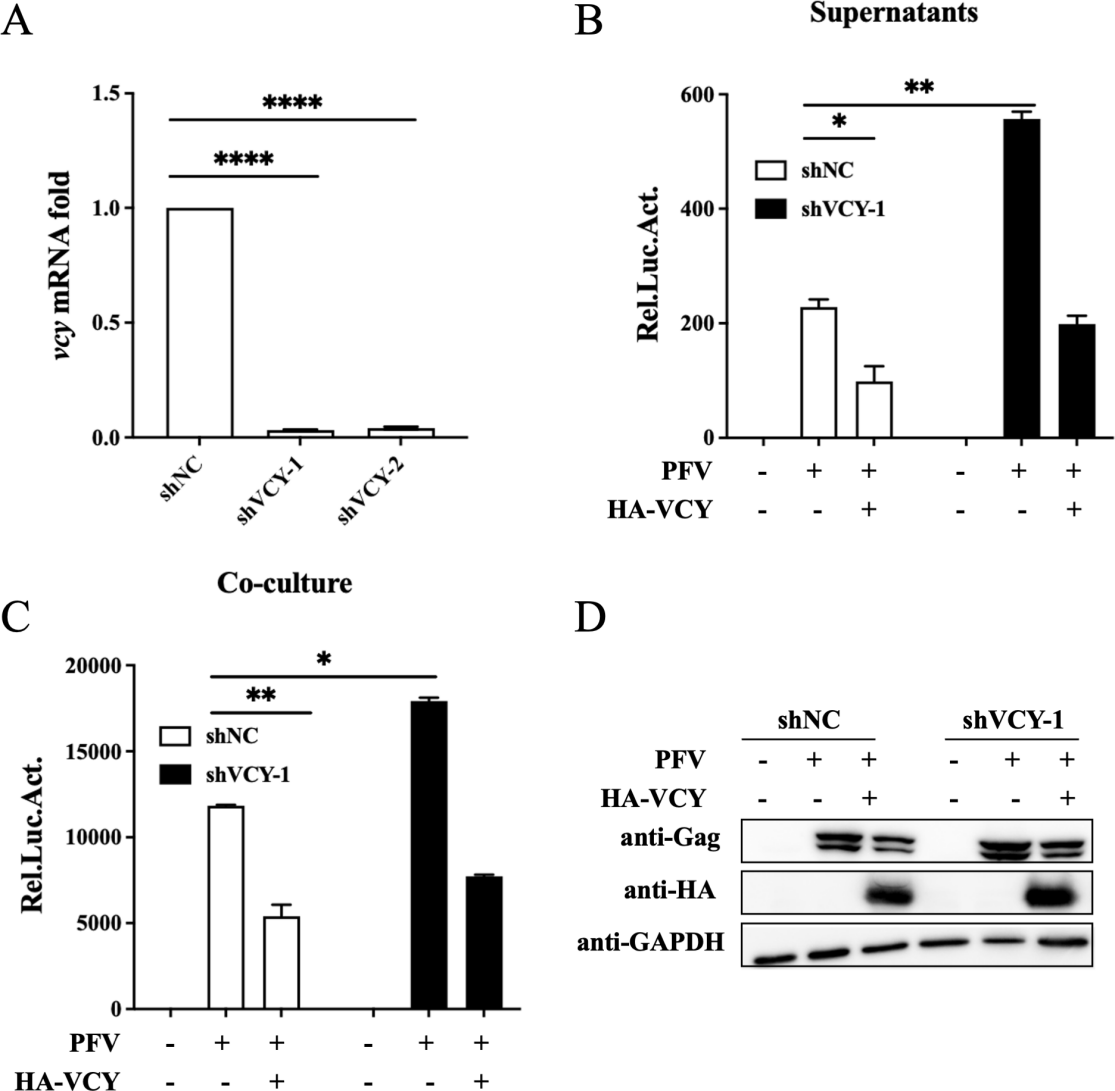
Knockdown of endogenous VCY promotes PFV replication. (**A**) HT1080 cells were infected with shNC, shVCY-1, and shVCY-2 viral particles. The VCY mRNA levels in HT1080 cells were measured using qRT-PCR. (**B through D**) HT1080-shNC and HT1080-shVCY-1 cells were infected with PFV (MOI = 0.5) after transfected with HA-VCY or an empty vector for 8 h. At 48 h post-infection, 600 µL of the supernatants (**B**) or 1/10 infected HT1080 cells (**C**) were incubated with PFVL cells, and the luciferase activity was measured 48 h later. (**D**) The rest of the infected cells were lysed for western blotting. ^*^P < 0.05, ^**^P < 0.01, ^****^P < 0.0001. All the data are representative of three independent experiments with triplicate samples. (^**^P < 0.01, ^***^P < 0.001).

Two specific shRNAs targeting VCY were synthesized to create VCY knockdown cell lines. The results showed that the knockdown effect was more pronounced in shVCY-1 cells (Fig. 3A). To evaluate the effect of VCY knockdown on PFV replication, both VCY-knockdown and control cells were infected with PFV for 48 h. As shown in Fig. 3B through D, PFV replication levels were significantly higher in the knockdown cells compared to the control cells, indicating that endogenous VCY had an inhibitory effect on PFV replication. To further confirm this inhibitory effect, we re-expressed VCY in both VCY-knockdown and control cells and observed that this led to a downregulation of PFV replication, further verifying VCY’s inhibitory impact on PFV replication (Fig. 3B through D).

### Inhibition of PFV replication by VCY N-terminal domain

**Fig 4:**
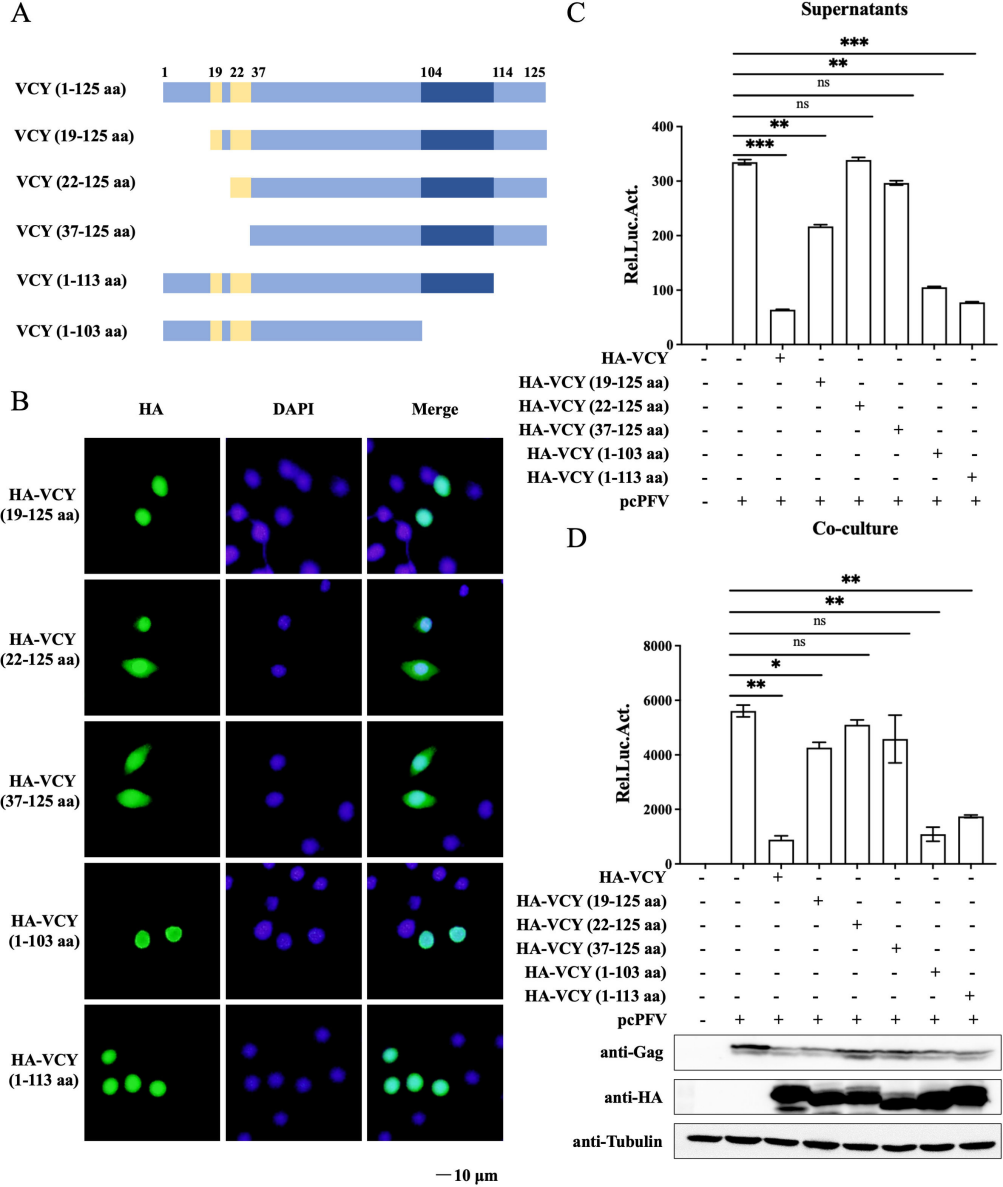
Inhibition of PFV replication by VCY N-terminal. (**A**) Schematic diagram of VCY and its truncated mutants. (**B**) HeLa cells were transfected with HA-VCY (19–125 aa), HA-VCY (22–125 aa), HA-VCY (37–125 aa), HA-VCY (1–103 aa), and HA-VCY (1–113 aa), respectively. At 48 h post-transfection, the cells were fixed, permeabilized, immunostained with anti-HA antibody, and then immunostained with FITC-labeled secondary antibody. Cell nuclei were stained with DAPI. The cells were observed under a confocal microscope. (**C, D**) HEK293T cells were transfected with pcPFV and HA-VCY or different truncations. After 48 h, 600 µL of the culture supernatants (**C**) or 1/20 transfected HEK293T cells (**D**) were co-cultured with PFVL cells. Luciferase activity was measured 48 h post-infection. The rest of the HEK293T cells were lysed for western blot analysis. All the data are representative of three independent experiments with triplicate samples. (^*^P < 0.05, ^**^P < 0.01, ^***^P < 0.001, and ns for *P* > 0.05).

To determine the critical domain of VCY responsible for inhibiting PFV replication, we constructed several plasmids according to previous report, including HA-VCY (19–125 aa), HA-VCY (22–125 aa), HA-VCY (37–125 aa), HA-VCY (1–113 aa), and HA-VCY (1–103 aa) (18) (Fig. 4A). Due to the presence of dual NLSs between amino acids 19–36 of VCY, the localization of each truncated mutant of VCY within cells was initially investigated. Immunofluorescence experiments revealed that the mutants HA-VCY (19–125 aa), HA-VCY (1–103 aa), and HA-VCY (1–113 aa), which contain the NLS regions, were predominantly located in the nucleus. In contrast, the mutants HA-VCY (22–125 aa) and HA-VCY (37–125 aa), which had the NLS regions partially or completely deleted, were found to be co-localized in both the nucleus and cytoplasm (Fig. 4B). Next, we explored the key VCY domains involved in its antiviral activity. The truncated expression plasmids or empty vectors were co-transfected with pcPFV into HEK293T cells. As shown in Fig. 4C and D, compared to the inhibitory effect of VCY on PFV replication, the inhibitory effect of overexpression of HA-VCY (19–125 aa) on PFV replication is relatively weakened, while overexpression of HA-VCY (22–125 aa) and HA-VCY (37–125 aa) did not inhibit PFV replication. Therefore, the key domain of VCY that inhibits PFV replication must be VCY N-terminal domain (1–21 aa).

**Fig 5:**
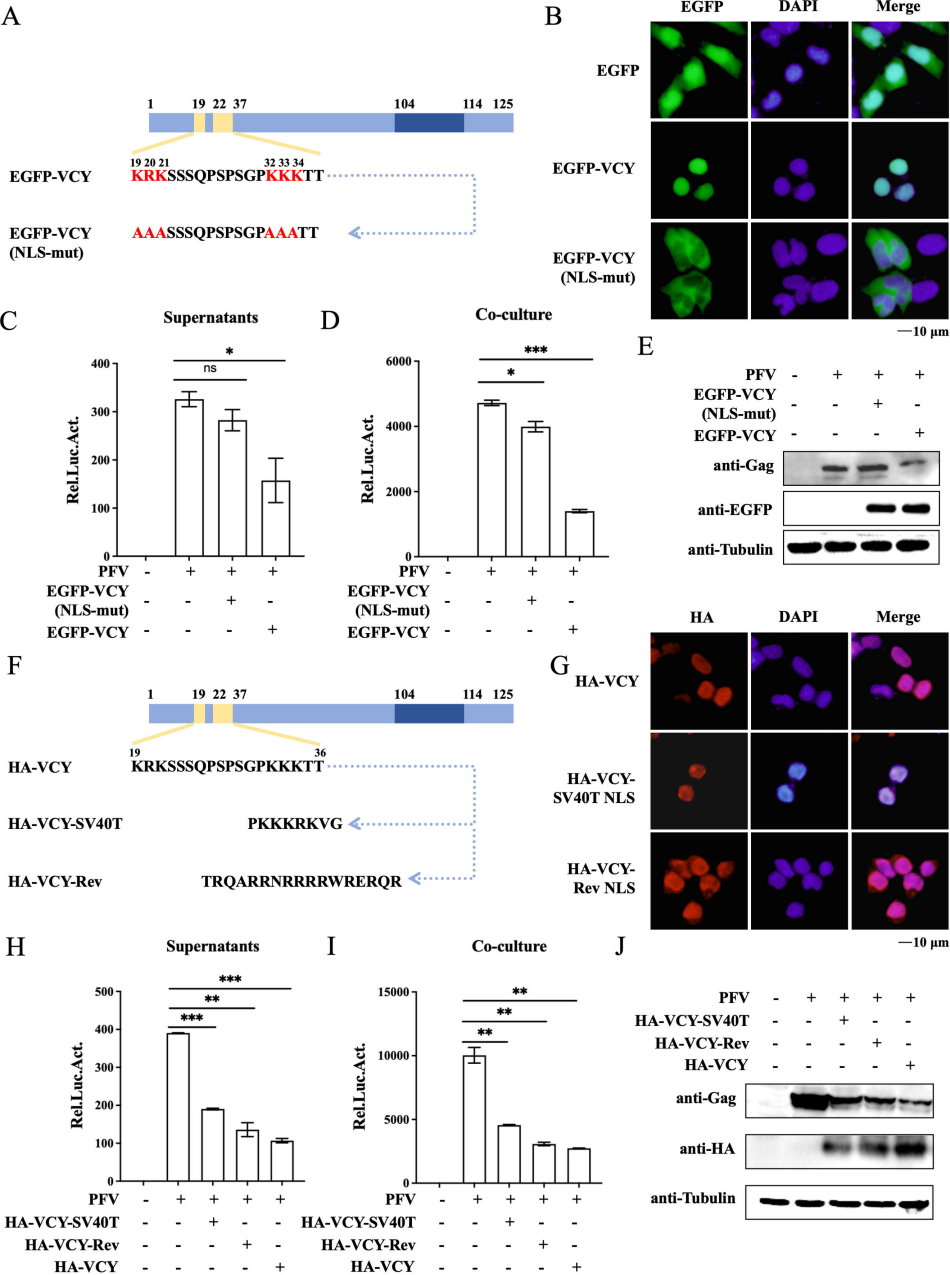
The nuclear localization of VCY is essential for the inhibition of PFV replication. (**A**) Schematic diagram of VCY and its NLS mutants. (**B**) HEK293T cells were transfected with EGFP-VCY, EGFP-VCY (NLS-mut), or an empty vector. At 48 h post-transfection, the cells were fixed and permeabilized. Cell nuclei were stained with DAPI. The cells were observed under a confocal microscope. (**C through E**) HT1080 cells were infected with PFV (MOI = 1) after being transfected with HA-VCY or different mutants for 8 h. After 40 h, 600 µL of supernatants (**C**) or 1/20 of infected HT1080 cells (**D**) were co-cultured with PFVL cells to determine viral titers by luciferase assay. (**E**) Western blotting was used to detect viral protein expression levels. All the data are representative of three independent experiments with triplicate samples. (^**^P < 0.01, ^***^P < 0.001). (**F**) Schematic diagram of VCY and its NLS mutants. (**G**) HEK293T cells were transfected with HA-VCY, HA-VCY-SV40T, and HA-VCY-Rev, respectively. At 48 h post-transfection, the cells were fixed, permeabilized, immunostained with anti-HA antibody, and then immunostained with TRITC-labeled secondary antibody. Cell nuclei were stained with DAPI. The cells were observed under a confocal microscope. (**H through J**) HT1080 cells were infected with PFV (MOI = 1) after transfected with HA-VCY or different mutants for 8 h. After 40 h, 600 µL of supernatants (**H**) or 1/10 of infected HT1080 cells (**I**) were co-cultured with PFVL cells to determine viral titers by luciferase assay. (**J**) Western blotting was used to detect viral protein expression levels. All the data are representative of three independent experiments with triplicate samples. (^**^P < 0.01, ^***^P < 0.001).

To explore whether the nuclear localization of VCY is necessary to inhibit PFV replication, we constructed a plasmid encoding a NLS-mutated VCY variant EGFP-VCY (NLS-mut) (Fig. 5A). Subcellular localization revealed that EGFP-VCY was localized in the nucleus, whereas the EGFP-VCY (NLS-mut) exhibited cytoplasmic localization (Fig. 5B). To determine whether alterations in the nuclear localization of VCY influence its capacity to inhibit PFV replication, HT1080 cells were transfected with EGFP-VCY or EGFP-VCY (NLS-mut) before infecting them with PFV virus stock solution. As shown in Fig. 5C through E, EGFP-VCY (NLS-mut) failed to inhibit PFV replication, indicating that VCY-mediated inhibition of PFV replication is dependent on its nuclear localization. We further investigated whether the NLS (19–36 aa) of VCY has sequence specificity. We replaced VCY’s BP NLS (19–36 aa) with a MP NLS (SV40T) and a ncNLS (Rev) to generate HA-VCY-SV40T and HA-VCY-Rev plasmid (Fig. 5F) (19, 24). Indirect immunofluorescence experiments demonstrated that both HA-VCY and variants localized to the nucleus (Fig. 5G). To investigate whether alterations in the VCY NLS affect its ability to inhibit PFV replication, we transfected plasmid HA-VCY and its variants into HT1080 cells and infected them with PFV virus stock solution. As shown in Fig. 5H through J, both variants were able to inhibit PFV replication to the same extent as HA-VCY. Together with these reports, we found that VCY-mediated inhibition of PFV replication is dependent on its nuclear localization, but not reliant on the sequence specificity of the NLS.

### VCY inhibits Tas from transactivating PFV LTR and IPs

**Fig 6:**
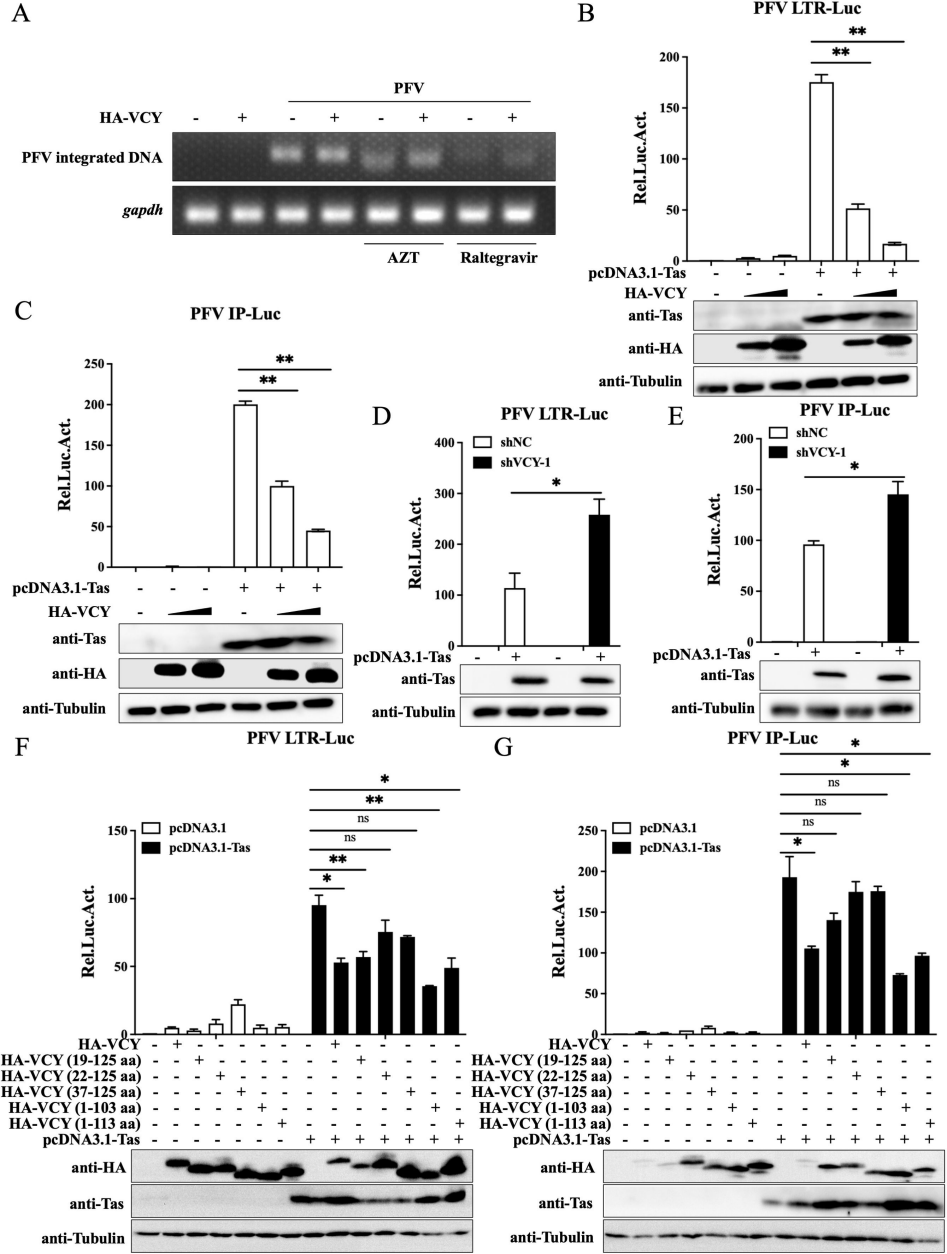
VCY inhibits Tas from transactivating PFV LTR and IPs. (**A**) Levels of integrated proviral DNA were measured using semiquantitative PCR. (**B, C**) HEK293T cells were transfected with PFV LTR-Luc (**B**) or IP-Luc (**C**) along with pCMV-β-Gal, Tas, and varying concentrations of HA-VCY. Luciferase activity was assessed after 48 h, followed by the collection of cell lysates for subsequent western blot analysis. (**D, E**) HT1080-shNC and HT1080-shVCY-1 cells were transfected with LTR-Luc (**D**) or IP-Luc (**E**) along with pCMV-β-Gal and Tas. Luciferase activity was assessed after 48 h, followed by the collection of cell lysates for subsequent western blot analysis. (**F, G**) HEK293T cells were transfected with LTR-Luc (**F**) or IP-Luc (**G**) along with pCMV-β-Gal, Tas, and HA-VCY or its different truncations. Luciferase activity was assessed after 48 h, followed by the collection of cell lysates for subsequent western blot analysis. All the data are representative of three independent experiments with triplicate samples. (^*^P < 0.05, ^**^P < 0.01 and ns for *P* > 0.05).

Additional studies were conducted to further investigate the mechanism by which VCY inhibits PFV replication. Genome integration is considered the boundary between early and late stages of retrovirus replication. Based on the principle of Alu-PCR, semiquantitative PCR was used to detect the integrated PFV genome. As shown in Fig. 6A, treatment with raltegravir (integrase inhibitor) and azidothymidine (AZT) (reverse transcriptase inhibitor) significantly inhibited PFV genome integration. However, VCY overexpression had no significant effect on PFV genome integration. These results suggested that VCY may exert its antiviral function during the late stage of PFV replication.

We next tested whether VCY affects PFV transcription. Since transcription in PFVs is mediated by Tas, we used PFV LTR-Luc and IP-Luc reporter plasmids to explore the effects of VCY on the basic transcriptional activity of PFV promoters and the transactivation ability of Tas. The results showed that VCY had no significant effect on the basic activity of the LTR and IPs; however, VCY dose-dependently inhibited Tas’s ability to activate these promoters (Fig. 6B and C). Moreover, endogenous VCY knockdown enhanced Tas-mediated transactivation of the LTR and IPs (Fig. 6D and E). These results suggested that VCY inhibits Tas’s transactivation of the PFV LTR and IPs.

We further explored the functional domain of VCY and found that overexpression of VCY and its truncated mutants alone did not affect the basal transcriptional activity of the LTR and IPs (Fig. 6F and G). However, when VCY truncated mutants were co-expressed with Tas, the inhibition of Tas transactivation by HA-VCY (22–125 aa) and HA-VCY (37–125 aa) was weakened (Fig. 6F and G), indicating that VCY exerts its inhibitory effect on PFV transcription through its N-terminal region, consistent with previous findings regarding the key domain of VCY in inhibiting PFV replication.

### VCY inhibits the function of Tas DNA-binding domain and activation domain

**Fig 7:**
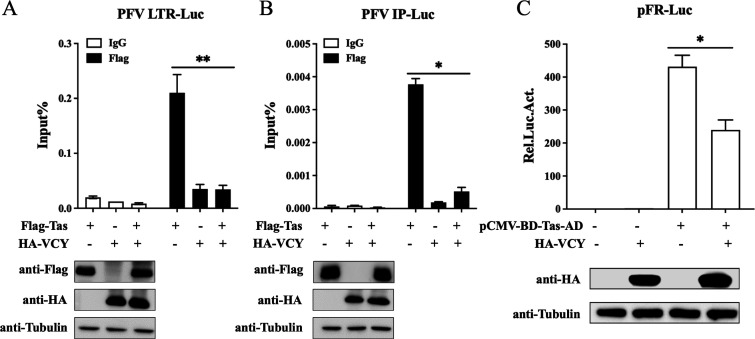
VCY inhibits the function of Tas DNA-binding domain and activation domain. (**A, B**) HEK293T cells were co-transfected with LTR-Luc (**A**) or IP-Luc (**B**) with HA-VCY, Flag-Tas, HA-VCY, and Flag-Tas, respectively. At 48 h post-transfection, cells were subjected to the ChIP assay. Subsequently, RT-PCR amplification was carried out to detect LTR (**A**) or IP (**B**) promoters in the immunoprecipitated chromatin fragments. The remaining cell lysates were collected for western blot analysis. (**C**) HEK293T cells co-transfected with pFR-Luc and pCMV-β-Gal, combined with pCMV-BD-Tas-AD, HA-VCY, HA-VCY, and pCMV-BD-Tas-AD, respectively. After 48 h, luciferase activity was measured. The remaining cell lysates were collected for western blot analysis. All the data are representative of three independent experiments with triplicate samples. (^*^P < 0.05, ^**^P < 0.01).

The above results suggested that VCY inhibits PFV transcription. During the regulation of PFV transcription, Tas must bind to the promoter to activate transcription after entering the nucleus. Therefore, chromatin immunoprecipitation (ChIP) was used to detect the effect of VCY on Tas’s DNA-binding ability. According to the results of RT-PCR, VCY impeded the binding of Tas to the LTR promoter (Fig. 7A). The protein levels of VCY and Tas were detected using western blotting (Fig. 7A). We also investigated whether VCY affects Tas binding to the IP, and the results showed that VCY also impeded Tas’s binding to the IP (Fig. 7B). Thus, VCY affects the DNA-binding ability of Tas. In addition to directly binding to PFV LTR or IPs through its N-terminal DNA-binding domain (80–210 aa), Tas activates transcription through its C-terminal activation domain (250–290 aa) (25). To further explore the influence of VCY on Tas’s transcriptional activation, we utilized a mammalian two-hybrid system with the plasmid pCMV-BD-Tas-AD, which combines the Tas activation domain with a heterologous DNA-binding domain. The results indicated that VCY inhibited the function of Tas activation domain (Fig. 7C).

### VCY-Tas interactions

**Fig 8:**
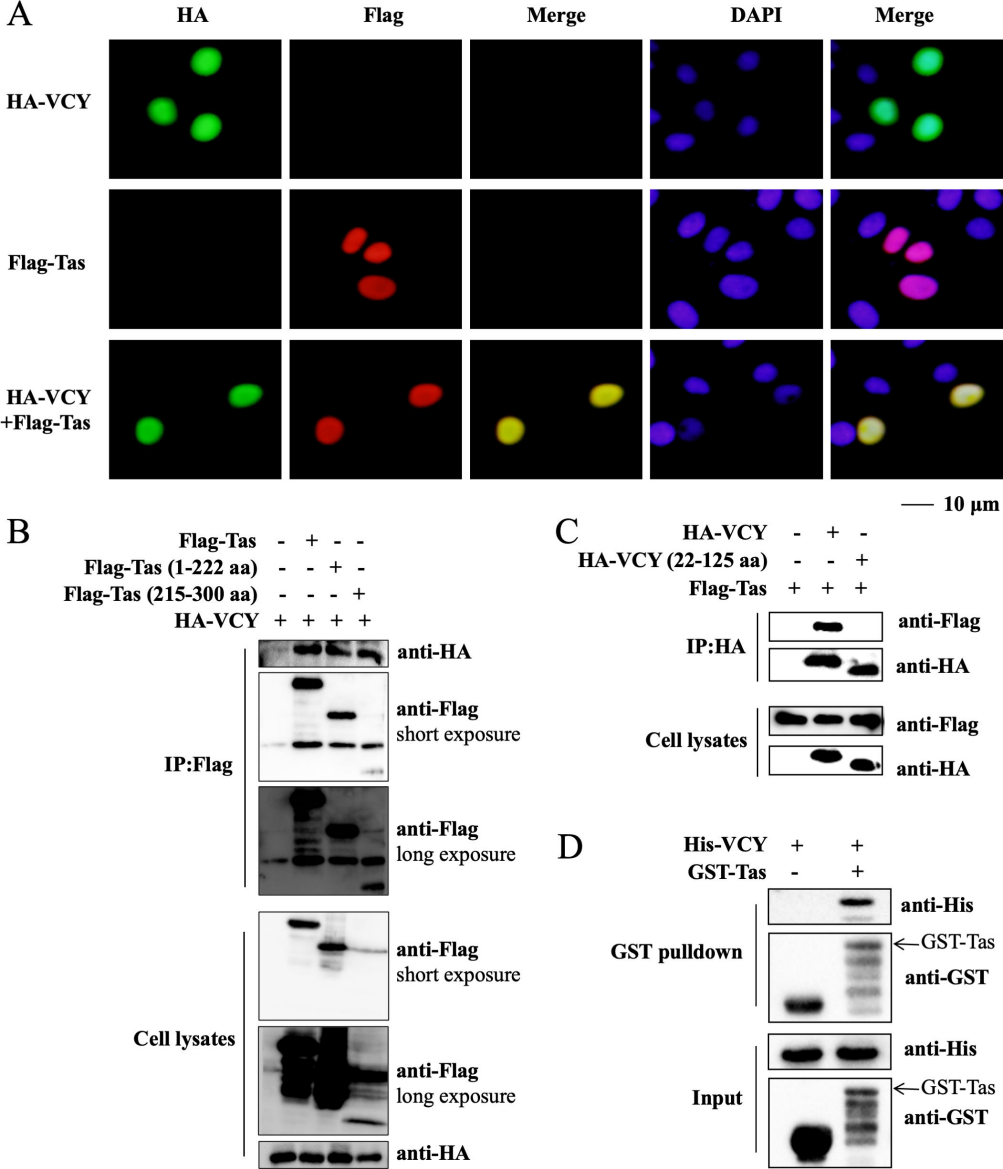
VCY-Tas interactions. (**A**) HeLa cells were co-transfected with HA-VCY, Flag-Tas, HA-VCY, and Flag-Tas, respectively. At 48 h post-transfection, the cells were fixed, permeabilized, immunostained with anti-HA antibody, and then immunostained with FITC-labeled secondary antibody or immunostained with anti-Flag antibody and then immunostained with TRITC-labeled secondary antibody. Cell nuclei were stained with DAPI. The cells were observed under a confocal microscope. (**B**) HEK293T cells were co-transfected with HA-VCY, HA-VCY and Flag-Tas, HA-VCY and Flag-Tas (1–222 aa), and HA-VCY and Flag-Tas (215–300 aa), respectively. The cells were collected at 48 h post-transfection and then used for Co-IP assay with anti-Flag antibody. (**C**) HEK293T cells were co-transfected with Flag-Tas, Flag-Tas and HA-VCY, and Flag-Tas and HA-VCY (22–125 aa), respectively. The cells were collected at 48 h post-transfection and then used for Co-IP assay with anti-HA antibody. (**D**) The purified recombinant His-VCY and GST-Tas protein were subjected to *in vitro* pull-down assay with glutathione sepharose, and the elution was examined by western blotting. The arrow indicates the GST-Tas protein.

We further examined whether VCY inhibits Tas’s functions through interaction in mammalian cells. Subcellular colocalization revealed that VCY co-localized with Tas in the nucleus (Fig. 8A). To determine the mechanism by which VCY affects Tas function, we co-transfected HEK293T cells with HA-VCY and Flag-Tas, along with a deletion mutant containing the DNA-binding domains (1–222 aa), and another mutant containing the activation domains (215–300 aa). Co-immunoprecipitation (Co-IP) results demonstrated the interaction between VCY and Tas, indicating that VCY interacts with both the Tas DNA-binding domain and activation domain (Fig. 8B). Reverse Co-IP results also indicated that VCY interacts with Tas, but the HA-VCY (22–125 aa) does not interact with Tas (Fig. 8C). Additionally, a GST pull-down assay using purified recombinant GST-Tas and His-VCY confirmed that VCY directly interacts with Tas (Fig. 8D). These observations suggest a role for VCY-Tas interactions in inhibiting PFV replication.

### Inhibition of BFV replication by VCY

**Fig 9:**
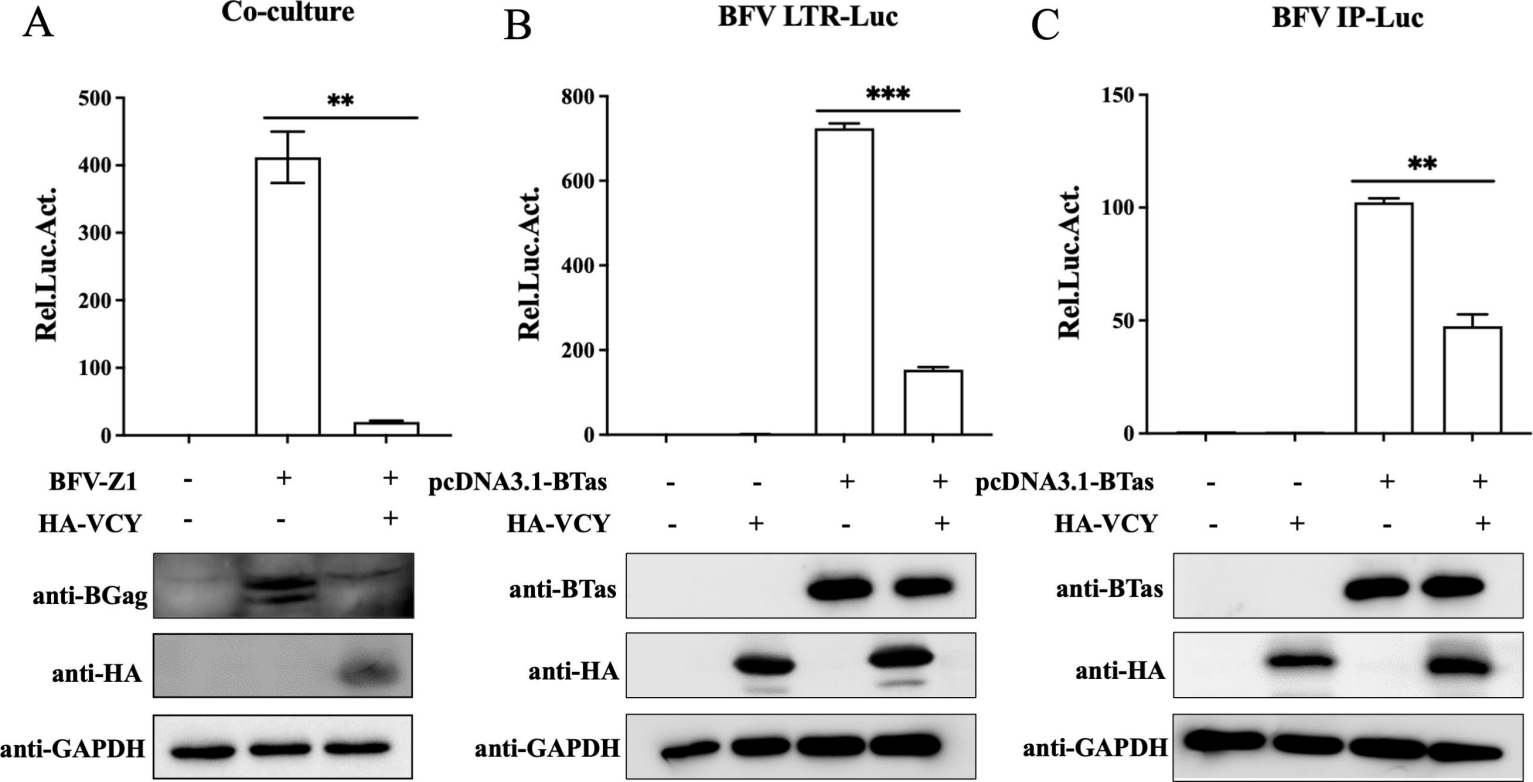
Inhibition of BFV replication by VCY. (**A**) HEK293T cells were transfected with BFV-Z1 and HA-VCY or an empty vector. After 48 h, 1/10 of infected cells were co-cultured with BFVL cells to determine viral titers by luciferase assay. The rest of the infected cells were lysed for western blotting. (**B, C**) HEK293T cells were transfected with BFV LTR-Luc (**B**) or IP-Luc (**C**) and pCMV-β-Gal, combined with BTas and HA-VCY. After 48 h, luciferase activity was measured. The remaining cell lysates were collected for western blot analysis. All the data are representative of three independent experiments with triplicate samples. (^**^P < 0.01, ^***^P < 0.001).

Next, we explored whether the antiviral effect of human VCY is specific to PFV. We assessed the effects of VCY on bovine FV (BFV), which also belongs to the *Spumaretrovirnae* subfamily. The infectious clone BFV-Z1 was co-transfected into HEK293T cells with plasmid HA-VCY. Since BFV can only establish infection through cell-to-cell transmission, we harvested the cells at 48 h post-transfection and incubated them with the BFV indicator cell line (BFVL). The BFV titer was determined by luciferase assays, and viral protein levels in the transfected cells were detected using western blotting. As shown in Fig. 9A, VCY overexpression significantly reduced the BFV titer, as well as cell BGag expression, indicating that VCY inhibits BFV replication. Given that VCY inhibits PFV replication by inhibiting PFV transcription, we investigated its effect on BFV transcription. As shown in Fig. 9B and C, VCY significantly inhibited the transactivation of BFV LTR and IPs by BTas. Therefore, VCY also inhibits BFV replication by acting at the transcriptional level.

## DISCUSSION

Recent years have seen a notable increase in emerging infectious diseases attributed to interspecies transmission resulting from viral mutations. Notable examples include avian influenza virus and severe acute respiratory syndrome, with pathogens from these outbreaks likely having zoonotic origins. FVs exhibit a broad tropism, capable of infecting a wide range of cell types, including epithelial and neural cells, highlighting their potential health threats that warrant further investigation (26). In this study, we demonstrate that VCY inhibits the transactivation of the PFV LTR and IPs through its interaction with Tas, impairing the functions of both the DNA-binding domain and activation domain of Tas.

Whether the weak disease association of PFV results from a balance between hijacking various cytokines to escape persecution by the host immune system and producing more progeny viruses to enhance survival remains unclear. The interaction between viruses and their hosts is characterized by a complex and dynamic interplay. For instance, PFV upregulated the transcription of dachshund family transcription factor 1 (DACH1), while urging its protein into SUMOylation, to eliminate the adverse effect of DACH1 overexpression on viral replication, thereby enhancing viral survival (27). Additionally, PFV infection increases SGK1 promoter activity via Tas, resulting in upregulation of endogenous SGK1 mRNA and protein expression (13). Conversely, PFV infection downregulates the expression of certain host genes to favor its own replication. For example, PFV infection induces abnormal expression of miRNAs targeting the zinc finger protein 219 (ZNF219)−3′UTR to downregulate ZNF219 expression (28). Furthermore, TRE2/BUB2/CDC16 domain family member 16 and recombinant V-rel reticuloendotheliosis viral oncogene homolog B also exhibit significant downregulation following PFV infection (29, 30). In the current study, we observed that among the 46 genes downregulated after PFV infection, the downregulation of *vcy* mRNA level was the most pronounced. Elucidating the precise mechanisms underpinning this phenomenon will be the focus of our forthcoming research endeavors.

Tas is primarily involved in the regulation of viral gene expression and is significant for both persistent and lytic infections of FVs (31). As a transcriptional regulator, Tas plays a critical role in viral replication. VCY inhibits PFV replication by interacting with the Tas protein, similar to other host factors like SGK1, promyelocytic leukemia protein (PML), Nmi, Pirh2, tripartite motif-containing protein 28 (Trim28), and prolactin regulatory element binding (PREB), all of which also suppress Tas’s transactivation function (11–13, 32, 33). However, the molecular mechanisms differ among these factors. SGK1 inhibits the transcriptional activation domain to recruit transcription factors, while PML prevents Tas from binding to the PFV promoter. Nmi blocks the nuclear entry of Tas, and Trim28 and Pirh2 reduce Tas levels via the proteasomal pathway (11–13, 32, 33). VCY not only inhibits the function of Tas’s DNA-binding domain, preventing its interaction with LTR or IPs, but also affects the function of its transcriptional activation domain. Similarly, the PREB impacts the functionality of both the Tas DNA-binding domain and transcriptional activation domain, thereby influencing Tas’s transactivation function and the recruitment of transcription factors (34).

The VCY genes are members of a gene family that also contains X-chromosomal members designated VCX, all with expression reported exclusively in male germ cells (18). Ayumu Taguchi et al. (23) found that VCY is a novel CTA, making it an ideal target for tumor immunotherapy and an adjunct to conventional cytotoxic cancer treatments. In the current study, we, for the first time, found the relationship between VCY and viruses, identifying VCY as a novel inhibitor of PFV replication. The N-terminal sequence of VCY contains a dual NLS, while the C-terminal region features a 10 amino acid repeat sequence (17). Another family member, VCX-A, has been identified as a decapping inhibitor, requiring the first 40 amino acids of its N-terminal region for activity. This suggests that all members of the VCX and VCY protein family, which share at least 94% identity in their amino-terminal halves, may also function as tissue-specific regulators of mRNA decapping and stability (35).

In viral infections, numerous nuclear localization proteins play a significant role in influencing viral replication. When these proteins are no longer localized in the nucleus, their ability to impact virus replication is compromised. For example, cleavage and polyadenylation specificity factor 6 (CPSF6) facilitates HIV-1 nuclear localization and infection, but an NLS mutant of CPSF6 does not promote HIV-1 infection (36). Similarly, transactive response DNA-binding protein (TARDBP/TDP-43) suppresses the production of HIV-1 viral particles, yet an NLS-deficient TARDBP/TDP-43 mutant fails to regulate HIV-1 infection (37). In addition, myxovirus resistance B (MxB) demonstrates the ability to inhibit HIV-1 infection, but when the N-terminal sequence of MxB is replaced with either the PY (Pro-Tyr) NLS or the arginine-rich NLS, it loses its capacity to suppress HIV-1 virus production (38). Consistent with these observations, VCY, recognized as an antiviral factor for PFV, possesses a BP NLS and is primarily located in the nucleus to carry out its function. Nevertheless, VCY-NLS mutants are ineffective in inhibiting PFV replication. In contrast to MxB, replacing VCY’s NLS with the MP NLS (SV40T) or a ncNLS (Rev) still leads to the inhibition of PFV replication. These findings suggest that the VCY-mediated inhibition of PFV replication relies more on its nuclear localization than on the specific sequence of the NLS. Additionally, to explore whether VCY’s antiviral properties exhibit broad-spectrum characteristics, we selected another member of the FV family, BFV, for initial investigation. Our preliminary findings indicate that VCY similarly inhibits BFV replication by targeting the transcriptional phase, suggesting that VCY may possess broad-spectrum antiviral properties.

In this study, we identified VCY as a novel protein associated with PFV that inhibits PFV replication by modulating the function of the transcription activator Tas. The functional domain of VCY responsible for this inhibitory effect is localized within the N domain. Furthermore, our data indicate that VCY influences the replication of both PFV and BFV. In conclusion, these results confirm the effects of VCY on retroviruses, demonstrating its antiviral spectrum and enhancing our understanding of its function, while indicating the broad scope of interactions between FVs and their hosts.

## MATERIALS AND METHODS

### Plasmids and antibodies

VCY was amplified using the cDNAs reverse-transcribed from HEK293T cells and cloned into pCMV-HA vector using primers as listed in Table 1. His-VCY, EGFP-VCY, and VCY truncations were generated based on HA-VCY using primers as listed in Table 1. Plasmid pLTR-Luc (39), pIP-Luc (39), and PFV full-length infectious clone (pcPFV) (40) were kindly provided by Maxine L. Linial (Division of Basic Sciences, Fred Hutchinson Cancer Research Center, Seattle, WA, USA). We purchased constructs of the pCMV-AD, pCMV-BD, and pFR-Luc from Stratagene (La Jolla, CA, USA) and pCMV-β-Gal from Invitrogen (Carlsbad, CA, USA). The constructs of pcDNA3.1-Tas (30), pFlag-Tas (30), pGST-Tas (41), the BFV infectious clone BFV-Z1 (42), pCMV-AD-Tas (1–220 aa), and pCMV-BD-Tas (223–300 aa) (13) were prepared as described previously. The sequences of all constructs were confirmed through sequencing. The antibodies used in this study include monoclonal mouse anti-HA (cat. no. H3663, Sigma-Aldrich, St. Louis, MO, USA), monoclonal rabbit anti-HA (cat. no. H6908, Sigma-Aldrich), monoclonal mouse anti-encoding glyceraldehyde-3-phosphate dehydrogenase (GAPDH) (cat. no. sc-47724, Santa Cruz Biotechnology, Dallas, TX, USA), monoclonal mouse anti-Flag (cat. no. F1804, Sigma-Aldrich), monoclonal rabbit anti-Flag (cat. no. F7425, Sigma-Aldrich), monoclonal mouse anti-tubulin (cat. no. sc-32293, Santa Cruz), monoclonal mouse anti-GST (cat. no. sc-138, Santa Cruz), monoclonal mouse anti-His (cat. no. sc-8036, Santa Cruz), monoclonal mouse anti-EGFP (cat. no. AE135, ABclonal Biotechnology, Wuhan, China), horseradish peroxidase (HRP)-conjugated goat anti-mouse IgG (cat. no. sc-2005, Santa Cruz), HRP-conjugated goat anti-rabbit IgG (cat. no. sc-2004, Santa Cruz), fluorescein isothiocyanate (FITC)-conjugated donkey anti-mouse IgG (cat. no. 715-095-150, Jackson Immuno Research Laboratories, West Grove, PA, USA), and tetramethyl rhodamine isocyanate (TRITC)-conjugated donkey anti-rabbit IgG (cat. no. 712-025-153, Jackson Immuno Research Laboratories). Polyclonal mouse sera against PFV Gag and Tas were prepared as described previously (30). The antibodies to BFV-Gag and BFV-Tas were also produced as described previously (43, 44). The chemical reagents used in this study were as follows: Protein A Beads (cat. no. 16-125, Millipore, Boston, MA, USA) and ClonExpress II One Step Cloning Kit (cat. no. C112-01, Vazyme, Nanjing, China).

**TABLE 1:** Primers used in this study

Application	Primer	Sequence (5′–3′)
Expression	HA-VCY-F	GCGAATTCATGAGTCCAAAGCCGAGAGCC
	HA-VCY-R	GCAAGCTTTCAGGGAGATAGGGGAGTAGATGGG
	HA-VCY (19–125 aa)-F	CCGGAATTCATGAAGAGGAAGTC
	HA-VCY (22–125 aa)-F	CCGGAATTCATGTCCTCCTCTCAG
	HA-VCY (37–125 aa)-F	CCGGAATTCATGAAGGTGGCCGAGAAG
	HA-VCY (1–103 aa)-R	TATAAGCTTTCAGGGGTCGTGCTGGGTC
	HA-VCY (1–113 aa)-R	TATAAGCTTTCATGGTTCCTCCAGCTC
	HA-VCY-SV40T-F	CCAAGGCCAAGGAGACAGGACCAAAGAAGAAGAGAAAGGTAAAGGTGGCCGAGAAGGGAG
	HA-VCY-SV40T-R	CTCCCTTCTCGGCCACCTTTACCTTTCTCTTCTTCTTTGGTCCTGTCTCCTTGGCCTTGG
	HA-VCY-Rev-F	CCAAGGCCAAGGAGACAGGAACCCGACAGGCCCGAAGGAATAGAAGAAGAAGGTGGAGAGAGAGACAGAGAAAGGTGGCCGAGAAGGGAG
	HA-VCY-Rev-R	CTCCCTTCTCGGCCACCTTTCTCTGTCTCTCTCTCCACCTTCTTCTTCTATTCCTTCGGGCCTGTCGGGTTCCTGTCTCCTTGGCCTTGG
	His-VCY-F	TCTGCCATGGCTGATATCGGATCCATGAGTCCAAAGCCGAGAGCC
	His-VCY-F	TTGTCGACGGAGCTCGAATTCGGGAGATAGGGGAGTAGATGGG
	EGFP-VCY-F	CCGCTCGAGATGAGTCCAAAGCCG
	EGFP-VCY-R	CCCAAGCTTGGGAGATAGGGGAGT
	EGFP-VCY(NLS-mut)-F	GACAGGAGCGGCGGCGTCCTCCTCTCAGCCGAGCCCCAGTGGCCCGGCGGCGGCGACTAC
	EGFP-VCY(NLS-mut)-R	GTAGTCGCCGCCGCCGGGCCACTGGGGCTCGGCTGAGAGGAGGACGCCGCCGCTCCTGTC
shRNA	shVCY-1-F	GATCCGGACTACCAAGGTGGCCGAGAATTCAAGAGATTCTCGGCCACCTTGGTAGTCCTTTTTTACGCGTG
	shVCY-1-R	AATTCACGCGTAAAAAAGGACTACCAAGGTGGCCGAGAATCTCTTGAATTCTCGGCCACCTTGGTAGTCCG
	shVCY-2-F	GATCCGGCCCGAAGAAGAAGACTACCATTCAAGAGATGGTAGTCTTCTTCTTCGGGCCTTTTTTACGCGTG
	shVCY-2-R	AATTCACGCGTAAAAAAGGCCCGAAGAAGAAGACTACCATCTCTTGAATGGTAGTCTTCTTCTTCGGGCCG
RT-PCR	GAPDH-F	AACAGCGACACCCACTCCTC
	GAPDH-R	CATACCAGGAAATGAGCTTGACAA
	GAPDH-promoter-F	TACTAGCGGTTTTACGGGCG
	GAPDH-promoter-R	TCGAACAGGAGGAGCAGAGAGCGA
	VCY-F	GGCCAAGGCCAAGGAGA
	VCY-R	ATGGGCGCCCCTTACTCA
	LTR-F	GTGAGATCGAATCTTTCCTTAAC
	LTR-R	CCGTACAATCTAGAAACTATCC
	IP-F	CGTGACTGTTAATGAAACAACG
	IP-R	GCTTTTGCTCTTTCAATCTGCTC
ALU-PCR	ALU1	TCCCAGCTACTGGGGAGGCTGAGG
	ALU2	GCCTCCCAAAGTGCTGGGATTACAG
	SpA	ATGCCACGTAAGCGAAACTTAGTATAATCATTTCCGCTTTCG
	Lambda	ATGCCACGTAAGCAAACT
	NestedR	GAAACTAGGGAAAACTAGG

### Cell culture and transfection

HEK293T, HT1080, HeLa, PFVL, and BFVL cells were grown in Dulbecco’s Modified Eagle’s Medium (DMEM) (cat. no. C11995500BT, Gibco, Grand Island, NY, USA) at 37 and 5% CO^2^ atmosphere. All cell culture media were supplemented with 10% fetal bovine serum (FBS) (cat. no. 10437036, Gibco, Grand Island, NY, USA), 100 g/mL penicillin (cat. no. 15070063, Gibco, Grand Island, NY, USA), and 100 g/mL streptomycin (cat. no. 15140122, Gibco, Grand Island, NY, USA). When the cells were grown to 70%–80% confluency, the plasmids or RNAs were mixed with polyethylenimine (cat. no. 24765-100, Polysciences, Warrington, PA, USA) in DMEM, followed by the incubation at room temperature for 10 min. The mixed solution is then transferred to the cell plates. At 6 h post-transfection, the DMEM medium was replaced by DMEM containing 10% FBS, and the cells were cultured for a further 18 h.

### PFV production and infection

HEK293T cells were transfected with 10 µg of pcPFV. After 48 h, the cells were centrifuged at 1,000 g for 10 min, and the supernatants were collected and filtered through a 0.45 µm pore-size filter membrane. The virus titer was determined by infecting PFVL cells as described previously (11), and then the multiplicity of infection (MOI) was calculated according to the method of Tai et al. (45). For virus infection, HT1080 cells were infected with PFV stock. After 48 h, the supernatants and infected cells were collected and cocultured with PFVL cells. The viral load was measured via the luciferase activity. The luciferase activity in the virus-infected group was divided by the levels observed in the mock group for normalization. The infected cells were also analyzed by western blotting using the indicated antibodies.

### Knockdown cell line generation

The knockdown cell lines were screened using a retrovirus vector system. HEK293T cells were transfected with 1 µg MLV-Gag-Pol, 0.5 µg VSV-G, and 1 µg shNC/shVCY plasmids. The sequence for the VCY shRNAs is listed in Table 1. After 48 h, the cells were centrifuged at 1,000 g for 10 min, and the supernatants were collected. HT1080 cells were infected with the pseudovirus and then subcultured in a selection medium containing 2 µg/mL puromycin. RT-PCR was used to detect the knockdown efficiency.

### Luciferase reporter assay

Cells were harvested 48 h after infection or transfection, and luciferase activity was measured using a Luciferase Reporter Assay System Kit (cat. no. E1501, Promega, Madison, WI, USA), according to the manufacturer’s instructions.

### Real-time PCR

Total RNA was extracted using TRIzol reagent (cat. no. 15596026, Invitrogen, Waltham, MA, USA; cat. no. AC0101, Sparkjade Biotechnology, Shandong, China) according to the manufacturer’s instructions. The RNA was reverse transcribed into cDNA using the PrimeScript RT Reagent Kit with gDNA Eraser (cat. no. RR092A, Takara, Ohtsu, Japan). Real-time PCR was detected by FastStart Universal SYBR Green PCR Master Mix (cat. no. B21203, Bimake, Houston, USA) method. Relative RNA levels were normalized with GAPDH mRNA. Primers are listed in Table 1. The amplification conditions were 95 for 3 min, followed by 40 cycles at 95 for 30 s and 65 for 30 s, and then a melt curve was acquired from 65℃ to 95℃. After RT-PCR, the melting curve was analyzed to ensure the specificity of the reaction. The data were analyzed by the 2^−ΔΔCt^ method.

### Alu-PCR

To detect the integration level of the virus, we transfected HT1080 cells with HA-VCY and the empty vector. The experimental group was treated with reverse transcriptase inhibitor AZT (10 µM) and integrase inhibitor raltegravir (10 µM) before virus infection (46, 47). After 2 h, PFV stock was added to infect the cells; after 30 h, the cells were collected, and total DNA was extracted using DNeasy Blood and Tissue Kit (cat. no. 69506, Qiagen, Duesseldorf, Germany), according to the manufacturer’s instructions. The integrated PFV DNA was measured by semiquantitative PCR with Alu-PCR primers. The Alu-PCR primers are listed in Table 1. The semiquantitative PCR cycling parameters were 95℃ for 5 min; 95℃ for 30 s, 55℃ for 30 s, 68℃ for 3 min, 30 cycles, 68℃ for 7 min.

### Immunofluorescence assay

HeLa or HEK293T cells were seeded into 12-well plates with glass coverslips and grew to 70%–80% confluency, followed by fixation with 4% paraformaldehyde at room temperature for 10 min. After being washed three times with PBS, cells were treated with 0.5% Triton X-100 for membrane permeabilization at room temperature for 15 min. Then the cells were blocked with 5% bovine serum albumin at room temperature for 2 h. The fixed cells were incubated with anti-HA or anti-Flag antibodies at room temperature for 2 h. After being washed three times with PBS, cells were incubated with FITC- or TRITC-conjugated goat secondary antibodies at room temperature for 45 min. After being washed three times with PBS, cells were stained with 2.5 µg/mL 4',6-diamidino-2-phenylindole (DAPI) at room temperature for 10 min. After three-time wash with PBS, the samples were observed under an Olympus X71 fluorescence microscope.

### Co-immunoprecipitation

Cell monolayers were washed with PBS and lysed with lysis buffer containing 50 mM Tris, 150 mM NaCl, 2 mM EDTA, 3% glycerol, 1% Triton X-100, and EDTA-free protease inhibitor cocktail tablets. After brief sonication, cell lysates were centrifuged at 4℃ and 12,000 g for 15 min. The supernatant was incubated with the indicated antibodies at 4℃ for 3 h. The interacting complexes were precipitated by incubation with Protein A Agarose at 4℃ for 3 h. This mixture was washed six times with the immunoprecipitation lysis buffer and then boiled with 2× SDS loading buffer for western blotting analysis.

### Recombinant protein purification and pull-down assay

The plasmid was transformed into *Escherichia coli* BL21 cells for prokaryotic expression. The expression of the fusion protein was induced with isopropyl-b-D-1-thiogalactopyranoside (cat. no. A100487, Sangon Biotech, Shanghai, China). Recombinant GST-Tas was purified with HyPur T GST 4FF PrePacked Gravity Column (cat. no. C600911, Sangon Biotech, Shanghai, China) and eluted from beads with elution buffer (50 mM Tris-HCl, pH 8.0, and 10 mM reduced glutathione). Recombinant His-VCY was purified with HyPur T Ni-NTA 6FF (His-Tag) PrePacked Gravity Column (cat. no. C600791, Sangon Biotech, Shanghai, China) and eluted from beads with elution buffer elution buffer (50 mM NaH_2_PO4, 300 mM NaCl, and 100 mM imidazole, pH 7.4). For pull-down assay, purified His-VCY protein was added to the purified GST-Tas or GST protein coupled to glutathione sepharose and incubated for 3 h at 4℃. Subsequently, the beads were washed, boiled, and subjected to western blot.

### ChIP assay

HEK293T cells were collected, and ChIP assay was performed using the EZ-ChIP Kit (cat. no. 17-371, Millipore, Burlington, MA, USA) according to the manufacturer’s protocol (13). Cells were transfected with corresponding plasmids. After 48 h, formaldehyde was added to the culture medium at a final concentration of 1% and kept at 37℃ for 10 min. After sonication, the chromatin was immunoprecipitated using specific antibodies or control antibodies. DNA was purified and subjected to RT-PCR to detect PFV LTR or IPs. GAPDH promoter was used as a control. Primers are listed in Table 1.

### Western blotting

Transfected or infected cells were harvested and lysed in radioimmunoprecipitation assay buffer at 4℃ for 30 min and then centrifuged at 12,000 g and 4 ℃ for 10 min. The supernatant was mixed with the loading buffer and then boiled to 100℃ for 10 min. Samples were separated by SDS-PAGE and transferred onto polyvinylidene difluoride membrane (cat. no. 10600023, GE, Healthcare). The membranes were blocked with 5% skim milk in Tris-buffered saline with Tween 20 (TBST) (25 mM Tris-HCl, 150 mM NaCl, and 0.1% Tween-20, pH 7.5) at room temperature for 45 min, followed by incubation with the indicated primary antibodies (1:1,000-1:3,000) at room temperature for 1.5 h. The membrane was washed with TBST three times and incubated with HRP-conjugated goat anti-rabbit-IgG antibody (1:8,000) or anti-mouse-IgG antibody (1:8,000) at room temperature for 45 min. The membrane was washed with TBST six times, and after that, HRP substrate was added to the membrane and used for signal detection.

### Statistical analysis

All statistical analyses were performed using the GraphPad Prism version 9.0 (GraphPad Software). The *P*-value was analyzed by Student’s *t*-test. *P* < 0.05 was considered to indicate statistical significance (^*^P < 0.05; ^**^P < 0.01; ^***^P < 0.001; ^****^P < 0.0001).

## Data Availability

No large primary data sets have been generated or deposited in external repositories.
